# Isolation, Identification, and Characterisation of a Novel ST2378 *Aeromonas hydrophila* Strain from Naturally Diseased Frogs, *Rana dybowskii*

**DOI:** 10.3390/pathogens13070552

**Published:** 2024-06-30

**Authors:** Ran Zhao, Jing Wang, Di Wang, Yanan Wang, Guo Hu, Shaowu Li

**Affiliations:** Key Laboratory of Aquatic Animal Diseases and Immune Technology of Heilongjiang Province, Heilongjiang River Fisheries Research Institute, Chinese Academy of Fishery Sciences, Harbin 150070, China; zhaoran@hrfri.ac.cn (R.Z.); wangjing@hrfri.ac.cn (J.W.); wangdi@hrfri.ac.cn (D.W.); ynanwang2024@163.com (Y.W.)

**Keywords:** *Aeromonas hydrophila*, *Rana dybowskii*, virulence genotype, antimicrobial susceptibility, MLST

## Abstract

In 2023, *Rana dybowskii* exhibiting characteristic skin ulcers were found on a farm in northeastern China. Subsequently, two dominant bacteria, *Aeromonas hydrophila* Rd001 and *Acinetobacter johnsonii* Rd002, were isolated from naturally infected *R. dybowskii*. Experimental infection confirmed that Rd001 was the primary pathogen responsible for the disease in *R. dybowskii*, with a mean lethal dose (LD_50_) of 6.25 × 10^2^ CFU/g. The virulence genotype of Rd001 was identified as *ser*+/*aha*+/*lip*+/*nuc*+/*hlyA*+/*aer*+/*alt*+/*ast*+/*act*+. Antimicrobial susceptibility testing indicated that Rd001 was sensitive to enrofloxacin, flumequine, and neomycin. MLST analysis showed that Rd001 belonged to a new sequence type of* A. hydrophila*, named ST2378. This study offered the first comprehensive investigation into the pathogenicity, virulence genotypes, antimicrobial resistance, and genetic traits of *A. hydrophila* isolated from *R. dybowskii*, providing a theoretical foundation for preventing and controlling *A. hydrophila* infections.

## 1. Introduction

*Rana dybowskii*, also known as Dybowski’s frog, is an amphibian species native to wooded regions in Korea, Japan, and northeastern China [1]. Amphibians, compared to aquatic and terrestrial animals, inhabit more variable environments, rendering them more susceptible to external environmental influences [2]. In recent decades, the wild populations of these frogs have declined steadily due to environmental degradation and increased capture for consumption and research purposes [3]. The intensive breeding of *R. dybowskii* started in the 20th century to meet consumer demands. However, with high-density culture and improper management, the development of this industry was severely limited by several bacterial diseases.

*Aeromonas hydrophila*, a ubiquitous waterborne Gram-negative bacterium [4], has been infecting a wide range of amphibians for over a century, including species like the tiger frog (*Rana rugulosa*) [5], mountain yellow-legged frog (*Rana muscosa*) [6], marsh frog (*Pelophylax ridibundus*) [7], and brown tree frog (*Litoria ewingii*) [8]. The pathogenicity of *A. hydrophila* is primarily determined by the presence of multiple virulence factors, such as aerolysin, serine protease, elastase, haemolysin, cytotoxic enterotoxins, lipase, and nuclease [9,10,11]. Variations in virulence genotypes among isolates can result in varying host symptoms and levels of pathology [12]. Therefore, it is crucial to investigate the presence of virulence factors and explore genetic diversity among different *A. hydrophila* isolates to comprehend their pathogenesis and epidemiology. However, there are limited data available regarding this aspect of *A. hydrophila* isolated from frogs.

In 2023, a disease characterised by skin ulcers emerged in *R. dybowskii* on a farm in Heilongjiang Province, China. Two dominant strains, *A. hydrophila* Rd001 and *Acinetobacter johnsonii* Rd002, were obtained from the infected *R. dybowskii*. The experimental challenge confirmed that Rd001 was the primary pathogen responsible for this disease. This study comprises the morphological and biochemical characterisation of the *A. hydrophila *Rd001, as well as the investigation of its virulence genotypes, drug resistance, and genetic properties in order to develop strategies for disease management.

## 2. Materials and Methods

### 2.1. Diseased R. dybowskii and Bacterial Isolation

Diseased *R*.* dybowskii* individuals (body weight of 5–10 g) were sampled in a frog farm located in Heilongjiang Province, China (128°43′1.641″ E, 47°45′16.032″ N) and used for bacterial isolation. The diseased *R. dybowskii* showed clinical signs of skin ulceration in the forelimbs revealing eroded muscles, and some of the affected *R. dybowskii* showed the same signs on the abdominal skin.

Five symptomatic diseased *R. dybowskii* were selected for bacterial strain isolation. Following euthanasia by double pithing, the frogs were dissected post skin cleansing with 75% ethyl alcohol. Bacteria were obtained from various organs of each frog (kidney, spleen, and external lesions) using a sterile loop, then cultured on Tryptose Soya Agar (TSA) and incubated at 28 °C for 24 h. The dominant bacteria were screened and re-inoculated on TSA and incubated at 28 °C for 24 h. Single colonies were then picked and inoculated into Tryptose Soy Broth (TSB) medium at 28 °C for 18 h, and then stored in TSB medium containing 20% (*v*/*v*) sterile glycerol at −80 °C. The dominant isolates were labelled as Rd001 and Rd002.

### 2.2. Physiological and Biochemical Characterisation

The purified Rd001 and Rd002 were revived from cryostock into TSA plate and incubated at 28 °C for 24 h. The Gram staining results were observed. Physiological and biochemical indices of this strain were subsequently measured using BD Phoenix M50 reference identification analyser (BD Biosciences, NJ, USA).

### 2.3. Sequencing and Analysis of 16S rRNA and gyrB Genes

Genomic DNA of Rd001 and Rd002 were extracted using a commercial Kit (TianGen, Beijing, China). The 16S rRNA gene from DNA templates was amplified using the universal primer 27F:5′-AGAGTTTGATCCTGGCTCAG-3′ and 1492R:5′-GGCTACCTTGTTACGACTT-3′. The *gyrB* gene from DNA templates was amplified using the universal primer gyrB-F:5′-GAAGTCATCATGACCGTTCTGCA(TC)GC(TCAG)GG(TCAG)GG(TCAG)AA(AG)TT(TC)GA-3′ and gyrB-R:5′-AGCAGGGTACGGATGTGCGAGCC(AG)TC(TCAG)AC(AG)TC(TCAG)GC(AG)TC(TCAG)GTCAT-3′. PCR was performed on a thermal cycler (Biometra TAdvanced, Jena, Germany) with 50 µL of reaction mixture, containing 40 ng DNA template, 2 µL of each primer (10 µM), and 25 µL 2×PrimeSTAR Mix (Takara, Shiga, Japan), with DNase-free water comprising the remainder of the mixture. The amplicons were detected and sequenced by Genesoul Technology Company (Harbin, China). BLAST searches of sequences were performed through the NCBI website. Phylogenetic trees were constructed using the neighbour-joining method in MEGA 7.0 software.

### 2.4. Experimental Infections

#### 2.4.1. *R. dybowskii* Husbandry

Healthy *R. dybowskii* (with average body weight of 5.36 ± 1.75 g) were purchased from a frog farm in Heilongjiang Province and transported to our laboratory. Laboratory conditions simulated the natural environment in the wild with water and gravel (20 ± 5 °C), and specimens were temporarily incubated for 7 d before being used for experimental infections. The *R*.* dybowskii* were fed with breadworms twice a day. Before the infection experiment, five *R*.* dybowskii* were randomly selected for bacteriological examination to verify the absence of the pathogen.

#### 2.4.2. Determination of Median Lethal Dose

In pre-challenge experiment, 12 *R. dybowskii* were divided into two groups and Rd001 and Rd002 suspensions at a concentration of 1 × 10^8^ CFU/mL were prepared for abdominal subcutaneous injection at a dose of 100 μL/*R. dybowskii*.

Seventy *R. dybowskii* were divided equally into 7 groups. The isolate Rd001 was prepared and suspended in sterile PBS buffer at the concentrations from 1 × 10^3^ to 1 × 10^8^ CFU/mL. *R. dybowskii* in 6 groups were intraperitoneally injected with the 6 concentrations of Rd001 suspensions at a dose of 100 μL/*R. dybowskii*. The remaining one group was injected with the same volume of sterile PBS as control. Following the challenge, the *R. dybowskii* were kept in the simulated environment and fed with breadworms twice a day. Mortality and clinical signs of *R. dybowskii* in each group were recorded at 9 am and 5 pm daily for 7 d. The mean lethal dose (LD_50_) of Rd001 was calculated by the improved Karber’s method in IBM SPSS Statistics version 19. The kidney of the infected *R. dybowskii* were subjected to the re-isolation of tested bacteria.

#### 2.4.3. Single Infection and Co-Infection

Thirty *R. dybowskii* were divided equally into 3 groups including a single infection group, a co-infection group, and a control group. In the single infection group, *R. dybowskii* were injected with 100 μL of Rd001 bacterial suspensions (6.09 × 10^4^ CFU/mL). In the co-infection group, injections consisted of 100 μL of Rd001 bacterial suspension (6.09 × 10^4^ CFU/mL) and 100 μL of Rd002 bacterial suspension (1 × 10^8^ CFU/mL). The control group *R. dybowskii* received 100 μL of sterile PBS injections. Following the challenge, the *R. dybowskii* were cultured as previously described. Mortality and clinical signs of *R. dybowskii* in each group were recorded at 9 am and 5 pm daily for 7 d.

### 2.5. Analysis of Antimicrobial Susceptibility

The broth microdilution method was used to determine the MICs of 8 antimicrobial agents for Rd001 isolate [13]. Ninety-six-well microplates with 8 antimicrobial agents were purchased from Fosundiagnostics (Shanghai, China). The concentrations of 8 antimicrobial agents were enrofloxacin (0.015–32 μg/mL), flumequine (0.125–256 μg/mL), thiamphenicol (0.25–512 μg/mL), florfenicol (0.25–512 μg/mL), sulfamonomethoxine (1–1024 μg/mL), doxycycline (0.06–128 μg/mL), trimethoprim/sulfamethoxazole (0.06/1.2–64/1216 μg/mL), and neomycin (0.125–256 μg/mL), respectively.

The Rd001 isolate was cultured on Mueller–Hinton (MH) agar and placed in a 28 °C incubator for 24 h. The 0.5 McFarland bacterial suspension was created in sterile saline, diluted to approximately 5 × 10^5^ CFU/mL with MH broth, and 50 µL of this suspension was dispensed into individual wells of the microplate. Two wells were added with bacterial suspension only and sterile MH broth only for positive and negative controls, respectively. The microplate was incubated at 28 °C for 24 h. *Escherichia coli *ATCC 25922 and *Staphylococcus aureus* ATCC 29213 were used as controls, and all incubated at 37 °C. The MIC was determined by observing the lowest antimicrobial concentration that inhibited the visible growth of the bacterium. MICs obtained are interpreted as sensitive, intermediate, or resistant, based on the criteria listed in the CLSI MIC standard [14].

### 2.6. Screening of Virulence Genes

The presence of 9 virulence genes [aerolysin (*aer*), serine protease (*ser*), elastase (*aha*), haemolysin (*hlyA*), cytotonic enterotoxins (*act*, *alt*, *ast*), lipase (*lip*), nuclease (*nuc*)] were analysed by PCR, and the reaction parameters are summarised in Table 1. The PCR products were separated on a 1% agarose gel that was stained with ethidium bromide (10 μg/mL) in Tris-acetate-EDTA buffer (TAE) using a Power Pac universal Electrophoresis unit (Bio-Rad, CA, USA). The visualisation of the results was carried out using a UV Transilluminator (Bio-Rad, CA, USA) and Quantity-One v4.6.

### 2.7. Multi Locus Sequence Typing (MLST)

MLST was performed through the amplification of *A. hydrophila* housekeeping genes (*gyrB*, *groL*, *gltA*, *metG*, *ppsA*, *recA*) [21]. The primers used for the PCR were obtained from the PubMLST-*Aeromonas* spp. database [22]. The PCR amplification of six pairs of housekeeping genes was performed, and the raw data obtained from sequencing were visualised using DNAman (DNAman v9.0, Lynnon Biosoft, USA) for auditing and splicing. The verified sequences for each of the Rd001 housekeeping genes were analysed within the PubMLST *Aeromonas* spp. database to assign allelic numbers and defined sequence types (STs). All sequence types are available in the PubMLST database.

The six housekeeping genes of Rd001 were concatenated in the order *gyrB*-*groL*-*gltA*-*metG*-*ppsA*-*recA*, and the ITOL function in PubMLST was applied to compare the isolates in multiple sequences with other type strains in the database. The neighbour-joining method of Interactive Tree of Life v.4 programme (https://itol.embl.de/, accessed on 11 March 2024) was used to construct the evolutionary tree.

## 3. Results

### 3.1. Clinical Examination

**Figure 1:**
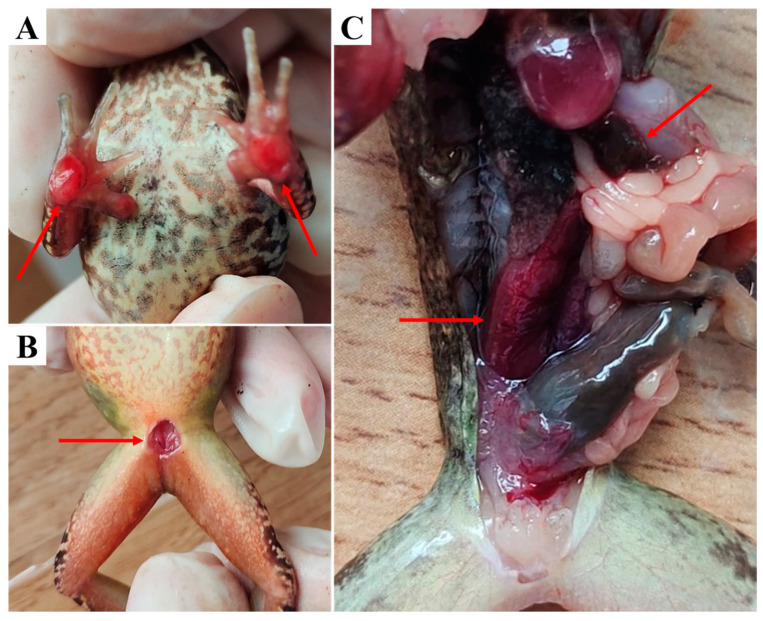
Clinical signs in diseased *R*.* dybowskii*. (**A**) Skin ulceration of the forelimbs, revealing corroded muscles. (**B**) Abdominal skin was broken and muscles are congested. (**C**) The kidney and spleen were congested and swollen.

Diseased *R. dybowskii* from the farm showed reduced activity, reduced feeding and hiding under shelters. Clinical examination showed that the frogs had broken skin on their forelimbs, exposing ulcerated muscles, some also displaying similar signs on the abdomen. Autopsy of the diseased frogs indicated congested and swollen kidney, as well as congested spleen (Figure 1).

### 3.2. Physiological and Biochemical Characteristics

Rd001 and Rd002 are both Gram-negative bacteria. Rd001 exhibits similar physiological and biochemical responses to *A. hydrophila* ATCC 35654. While they can both hydrolyse Esculin, Rd001 produces acid from Sucrose, D-Galactose, Maltulose, D-Gluconic acid, and Methyl-B-Glucosid; uses Citrate as its sole carbon source; cannot hydrolyse Urea; and cannot ferment Sorbitol, L-Rhamnose, and D-Melibiose. Notably, Rd001 is able to utilise Adonitol but lacks the ability to ferment L-Arabinose (Table 2).

Rd002 exhibits similar physiological and biochemical responses to *A. johnsonii* ATCC17909, which can use Adonitol and Citrate as its sole carbon source; cannot hydrolyse Esculin and Urea; and cannot to ferment Sorbitol, Sucrose, D-Galactose, Maltulose, L-Rhamnose, D-Gluconic acid, D-Melibiose, L-Arabinose, and Methyl-B-Glucosid (Table 2).

### 3.3. Sequence Analysis of the 16S rRNA and gyrB Genes

**Figure 2:**
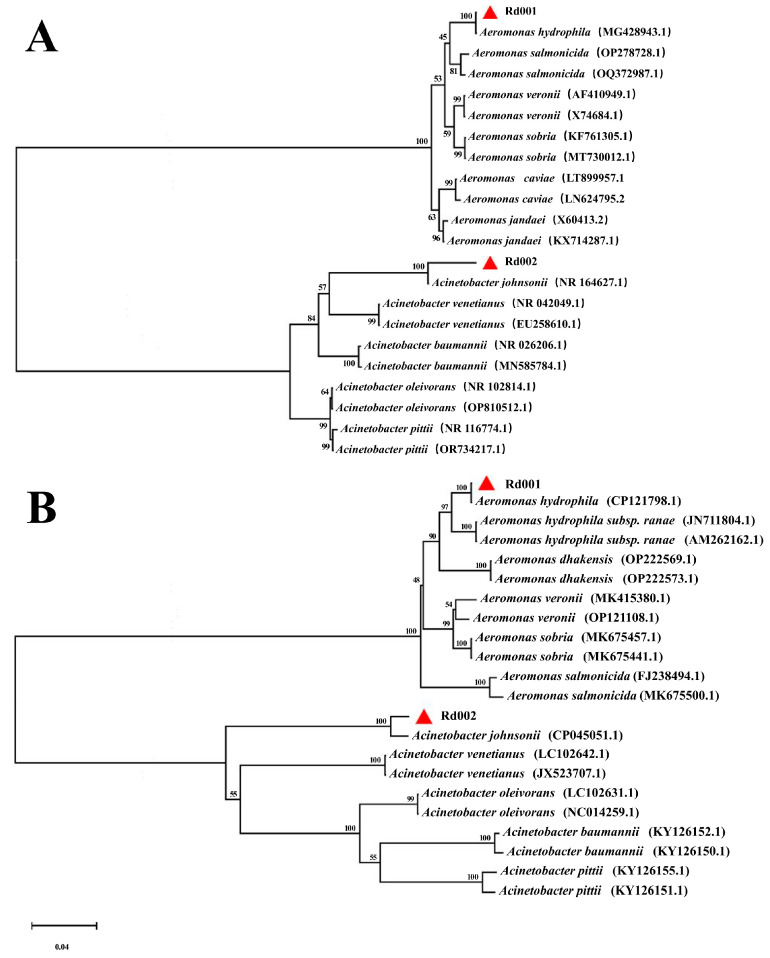
Molecular identification of the isolate Rd001 and Rd002. (**A**) Neighbour-joining phylogenetic tree based on the 16S rRNA gene sequence. (**B**) Neighbour-joining phylogenetic tree based on the *gyrB* gene sequence.

The *16S rRNA* gene sequences of Rd001 and Rd002 obtained by PCR amplification have been uploaded at NCBI with GenBank accession numbers PP094559.1 and PP658225, respectively. BLAST results showed that isolate Rd001 shares 100% identity with the *A. hydrophila* (MG428943.1), and isolate Rd002 shares 99.7% identity with the *A. johnsonii *(NR164627.1). The *gyrB* gene sequences of Rd001 and Rd002 obtained by PCR amplification have been uploaded at NCBI with GenBank accession numbers PP894756 and PP894757, respectively. BLAST results showed that isolate Rd001 shares 100% identity with the *A. hydrophila* (CP121798.1) and isolate Rd002 shares 97.8% identity with the *A. johnsonii *(CP045051.1). Based on the phylogenetic trees constructed using the 16S rRNA and *gyrB* genes sequences (Figure 2), isolate Rd001 was grouped with recognised strains of *A. hydrophila*, while Rd002 was associated with *A. johnsonii*. The results of BLAST and phylogenetic analysis confirmed that Rd001 is* A. hydrophila *and Rd002 is* A. johnsonii*.

### 3.4. Experimental Infections

#### 3.4.1. Determination of Median Lethal Dose

**Figure 3:**
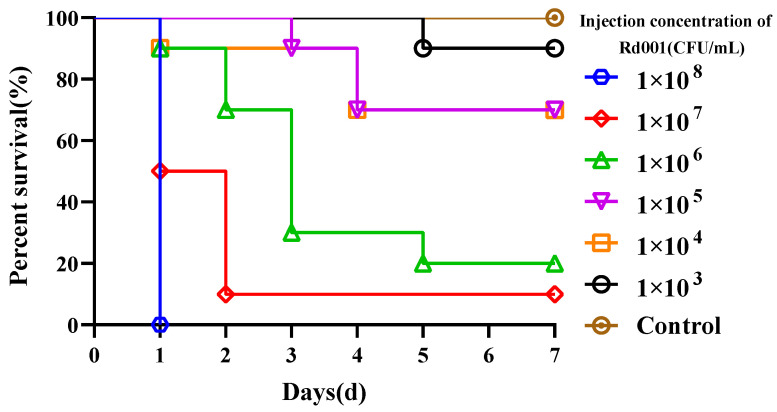
Cumulative mortalities of *R*.* dybowskii* challenged with different concentrations of Rd001.

In a pre-challenge experiment, *R*.* dybowskii* injected with 1 × 10^8^ CFU/mL Rd001 reached 100% mortality within 24 h, whereas *R*.* dybowskii* injected with the same dose of Rd002 did not die within 7 d. Subsequently, we determined the LD_50_ of Rd001 on the *R*.* dybowskii*, and there was no mortality in the control group during the experimental period, and the cumulative mortality rates of the frogs injected with Rd001 at concentrations ranging from 1 × 10^8^ to 1 × 10^3^ CFU/mL were 100%, 90%, 80%, 30%, 30%, and 10%, respectively (Figure 3). The LD_50_ of Rd001 was calculated as 6.09 × 10^4^ CFU/mL (6.25 × 10^2^ CFU/g). In addition, the bacterium was re-isolated from the kidney of artificially infected *R*.* dybowskii*.

#### 3.4.2. Single Infection and Co-Infection

**Figure 4:**
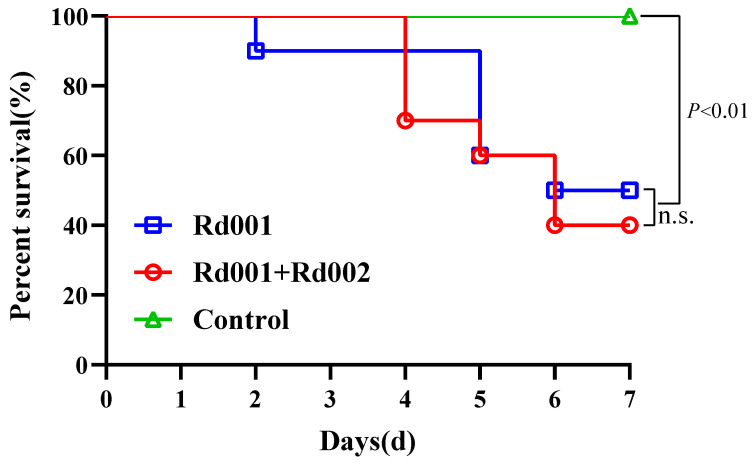
Cumulative mortalities in single-infected (Rd001), co-infected (Rd001 + Rd002), and control group of *R. dybowskii* at different time points. Comparison of survival curves were performed by a log-rank (Mantel–Cox) test using GraphPad Prism v9.3.1.

Throughout the experimental period, no deaths were recorded in the control group. In the co-infection group (Rd001 + Rd002), mortality began on the 3rd day post-challenge, with a cumulative rate of 60% by the 7th day. In the single-infection group (Rd001), mortality commenced on the 2nd day, and by the 7th day, the cumulative mortality rate reached 60% (Figure 4). There was no significant difference in the mortalities between the single-infected and co-infected frogs (*p* > 0.05).

### 3.5. Antimicrobial Susceptibility and Virulence Factors

The results of antimicrobial susceptibility of isolate Rd001 were shown in Table 3. The isolate Rd01 was sensitive to enrofloxacin, flumequine, and neomycin with a MIC value of 0.25 μg/mL, 2 μg/mL, and 1 μg/mL, respectively, and intermediated to doxycycline with a MIC value of 8 μg/mL, whereas it exhibited reduced susceptibility to thiamphenicol, florfenicol, sulfamonomethoxine, and trimethoprim/sulfamethoxazole, with MIC values of 512 μg/mL, 512 μg/mL, 512 μg/mL, and 4/76 μg/mL, respectively (Table 3).

**Figure 5:**
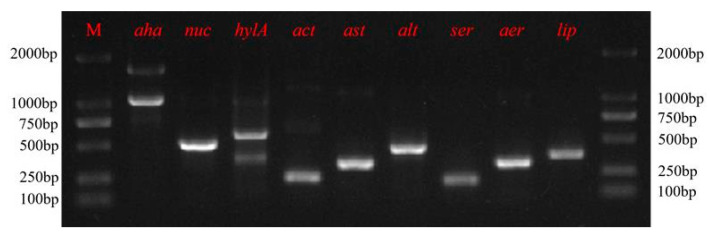
PCR amplification for nine virulent genes of Rd001. Lane M: DNA marker.

As shown in Figure 5, the isolate Rd001 was positive for *aer*,* ser*, *alt*, *ast*, *act*, *hlyA*, *nuc*, *aha*, and *lip*, as seen through the PCR validation of the virulence factors.

### 3.6. MLST Analysis of Rd001

Six housekeeping genes in Rd001 for MLST analysis were successfully amplified. The allelic profile of Rd001 (*gyrB*-804, *groL*-369, *gltA*-1041, *metG*-301, *ppsA*-269, *recA*-1130) was obtained by sequencing, splicing, and matching. It turned out that no sequence type matching this allelic profile existed in the PubMLST database, so we uploaded our original data and obtained a new sequence type number: ST2378 (Table 4).

**Figure 6:**
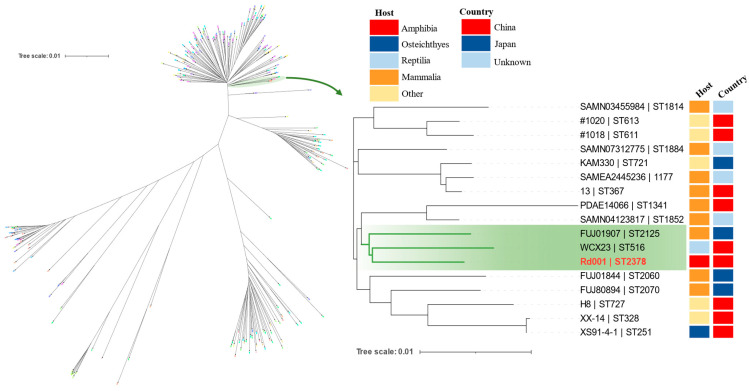
Neighbour-joining tree constructed from the tandem nucleotide sequences (*gltA*-*groL*-*gyrB*-*metG*-*ppsA*-*recA*) of six housekeeping genes from 214 *A. hydrophila* strains (**Left**). An evolutionary tree of the 17 most closely related strains of Rd001/ST2378 with a dataset including strain labelling, host, and country is presented (**Right**).

A systematic phylogenetic tree was constructed for ST2378, confirming its phylogenetic position within *A. hydrophila* PubMLST. Among the 16 strains closely related to it, 9 strains (56.25%) were isolated from mammals. Phylogenetic tree results revealed the clustering of ST2378 with ST516 and ST2125, with the closest phylogenetic relationship to ST516 isolated from reptilia in China (Figure 6).

## 4. Discussion

*A. hydrophila* is a prevalent pathogen in amphibians, capable of inducing clinical signs like red-leg syndrome and skin ulcers in frogs [25]. Recent reports on *A. hydrophila* have primarily focused on epidemiological investigations and host response mechanisms [26,27,28], neglecting research on pathogen virulence traits and genetics. This gap has impeded the progress in developing vaccines and control medications to a certain extent.

In this study, we identified the coexistence of *A. hydrophila* Rd001 and *A. johnsonii* Rd002 as dominant bacteria in naturally occurring lesions of *R. dybowskii*, with primary signs presenting as ulcers on the forelimbs and abdomen skin. Abdominal subcutaneous injection infection confirmed that Rd001 was the primary pathogen responsible for the disease of *R*.* dybowskii*, with a LD_50_ of 6.25 × 10^2^ CFU/g in 7 d. Based on the virulence characteristics of *Aeromonas* spp., Rd001 was classified as highly virulent [29].

Variances in disease signs and mortality resulting from distinct pathogenic* A. hydrophila* strains could stem from variations in virulence genotypes among isolates, with virulence genotyping recognised in multiple studies as a valuable tool for assessing the pathogenicity of *A. hydrophila* [30,31]. The Rd001 virulence genotype was identified as *ser*+/*aha*+/*lip*+/*nuc*+/*hlyA*+/*aer*+/*alt*+/*ast*+/*act*+, resembling the highly virulent *A. hydrophila* strains obtained from fish (*Tilapia zillii* and *Mugil cephalus*) in Egypt [32].

The *ser* and *aha* encode serine protease and elastase, respectively, both of which are extracellular enzymes that degrade epithelial mucous membranes and can lead to lesions in the skin and internal organs [33]. This contributes to infection establishment and evasion of the host’s initial defence mechanisms [34]. The *lip* encodes an extracellular lipase that enhances the severity of bacterial infection by modifying the host’s plasma membrane [35]. The *aer *and *hlyA* encode aerolysin and haemolysin, respectively, both of which are haemolytic toxins. Aerolysin, often present in pathogenic *Aeromonas* spp., induces osmotic cell lysis by binding to membrane-specific glycoprotein sites, leading to structural polymerisation [36]. Haemolysin is haemolytic and enterotoxic, binding to and lysing host erythrocytes [37]. Different enterotoxins encoded by the *act*, *act*, and *ast* can induce haemolysis and trigger the production of inflammatory mediators in macrophages and epithelial cells, leading to apoptosis, and elevate cAMP levels in intestinal epithelial cells, disrupting the water–electrolyte balance and causing diarrhoea [38,39].The synergistic action of multiple virulence factors in the strain Rd001 contributes to its survival, reproduction, and spread in the host. This action also leads to the destruction of host tissues and the suppression of the immune response, ultimately exacerbating the infection.

Bacterial drug resistance is a significant factor affecting the susceptibility of hosts to infection [40]. Rd001 showed reduced susceptibility to thiamphenicol, florfenicol, sulfamonomethoxine, and trimethoprim/sulfamethoxazole in an eight-antibiotic test, while being sensitive to enrofloxacin, flumequine, and neomycin. Enrofloxacin, with an MIC of 0.25 μg/mL, effectively inhibited Rd001 growth, thus emerging as the primary drug for the ongoing *R. dybowskii* outbreak. However, due to limited research on drug administration methods in amphibians and a lack of detailed pharmacokinetic studies, determining a reasonable dosage is challenging, necessitating further investigation.

MLST is frequently used to investigate the epidemiology, pathogenicity, and evolution of* A. hydrophila*, as well as to trace hosts and geographical origins [21,41]. In this study, the allelic profile of the identified Rd001 did not match the existing PubMLST data, leading to its classification as a novel sequence type: ST2378. The analysis of the phylogenetic tree constructed with existing *A. hydrophila* data from PubMLST shows that out of the 16 strains closely linked to ST2378, 9 strains (56.25%) are derived from mammals. Importantly, ST2378 is most closely related to ST516, which originates from China and is capable of inducing fatal diarrhoea in Chinese Moccasin (*Deinagkistrodon acutus*) [42]. Additionally, a high dose of ST516 can result in acute death in mice. Therefore, additional research is necessary to determine if ST2378 presents a public health risk to humans.

In general, *A. hydrophila* Rd001, isolated from the infected *R. dybowskii*, was identified as the primary pathogen with high virulence characteristics. MLST analysis suggested that Rd001 had a potential risk of infection in mammals, and relevant findings filled the gap in the genetics of *A. hydrophila* isolated from *R. dybowskii*. Given its significance as a major pathogen in frog infections, *A. hydrophila* requires urgent investigation for optimal drug delivery methods and pharmacokinetics, informed by current drug sensitivity data, and the implementation of infection control measures to limit its spread.

## Figures and Tables

**Table 1:** Primers for PCR detection of 9 virulence genes.

Gene	PCR Prime Sequence (5′→3′)	Product Size (bp)	Reference
*aer*	F: ACAGCCAATATGTCGGTGAAG	326	[15]
	R: TCACCTTCTCGCTCAGGC		
*ser*	F: CACCGAAGTATTGGGTCAGG	211	[16]
	R: GGCTCATGCGTAACTCTGGT		
*alt*	F:TGACCCAGTCCTGGCACGGC	442	[17]
	R:GGTGATCGATCACCACCAGC		
*ast*	F:TCTCCATGCTTCCCTTCCACT	331	[17]
	R:GTGTAGGGATTGAAGAGCCG		
*act*	F:AGAAGGTGACCACCACCAAGAACA	232	[17]
	R:AACTGACATCGGCCTGAACTC		
*hlyA*	F:GGCCGGTGGCCCGAAGATACGGG	592	[18]
	R:GGCGGCGCCGGACGAGACGGGG		
*nuc*	F:CAGGATCTGAACCGCCTCTATCAGG	504	[15]
	R:GTCCCAAGCTTCGAACAGTTTACGC		
*aha*	F:GGTATTGTATCCCGGCTCTGTT	1082	[19]
	R:CGGTCCATCGTCGTCCATCTTG		
*lip*	F: TCTTCTCCGACTGGTTCGG	382	[20]
	R: GTGCCAGGACTGGGTCTT		

**Table 2:** Physiological and biochemical characteristics of Rd001 and Rd002 isolates.

Characteristics	Rd001	*A. hydrophila* ATCC35654 [23]	Rd002	*A. johnsonii* ATCC17909 [24]
Gram stain	–	–	–	–
N-Acetyl-D-glucosamine	+	+	–	–
Adonitol	+	–	+	–
Citrate	+	+	+	–
Sorbitol	–	–	–	–
Sucrose	+	+	–	–
Glucose	+	+	–	–
D-Galactose	+	+	–	–
Maltulose	+	+	–	–
L-Rhamnose	–	–	–	–
D-Gluconic acid	+	+	–	–
D-Melibiose	–	–	–	–
L-Arabinose	–	+	–	–
Methyl-B-Glucoside	+	+	–	–
Urea	–	–	–	–
Esculin	+	+	–	–

**Table 3:** Drug susceptibility of Rd001 to eight antimicrobial drugs.

Group	Antibiotic	MIC (μg/mL)	Susceptibility
4-quinolones	Enrofloxacin	0.25	S
	Flumequine	2	S
Chloramphenicols	Thiamphenicol	512	R
	Florfenicol	512	R
Sulfonamides	Sulfamonomethoxine	512	R
	Trimethoprim/Sulfamethoxazole	4/76	R
Aminogly cosides	Neomycin	1	S
Tetracyclines	Doxycycline	8	I

Note: Susceptibility is designated as S = sensitive, I = intermediate, and R = resistant.

**Table 4:** Multi-locus sequence typing analysis of Rd001.

Isolate	Rd001
*gyrB*	804
*groL*	369
*gltA*	1041
*metG*	301
*ppsA*	269
*recA*	1130
Assigned ST	2378

## Data Availability

Data is contained within the article.
